# Ethical and social recognition of participation in drug clinical trials: French public views on financial compensation for participants

**DOI:** 10.1371/journal.pone.0343321

**Published:** 2026-08-24

**Authors:** Laure Peyro-Saint-Paul, Cathy Gaillard, Célia Berchi, Clément Gakuba, Charlotte Gourio, Guillaume Grandazzi, Michael Joubert, Sylvie Brucato, Clémence Bechade, Rémy Morello, INDEM Working Group

**Affiliations:** 1 Clinical Research Department, CHU Caen Normandy, Caen, France; 2 Caen Normandy University, Caen, France; 3 NIMEC (Normandie Innovation Marché Entreprise Consommation), Caen, France; 4 Comité de Protection des Personnes Nord Ouest III, Caen, France; Aix-Marseille Universite, FRANCE

## Abstract

**Background:**

Drug clinical trial participants should be fairly compensated for inconvenience while avoiding undue inducement. However, European regulations provide limited operational guidance on when compensation should be offered and how it should be determined. The INDEM3 project aims to develop a decision-support tool for sponsors and ethics committees, beginning with an assessment of exploratory public perspectives in France.

**Methods:**

An exploratory population-based survey was conducted using a self-administered questionnaire with an estimated completion time of approximately seven minutes. A minimum of 60 individuals was targeted, with or without chronic condition or prior research experience. The questionnaire assessed general views on financial compensation and responses to three fictional drug clinical trial scenarios varying in risk, burden, and direct benefit. Distribution occurred in healthcare settings and via social networks.

**Results:**

Between March 2024 and January 2025, 65 responses were collected. Most respondents (75%) supported financial compensation for participation in drug clinical trials. The most frequently cited criteria were exposure to a new investigational drug (65%), invasive procedures (63%) and experiencing income lost (63%), and absence of direct personal benefit (28%). For the scenarios, the proportion of respondents supporting compensation and the median proposed amounts were as follows: Scenario 1 (no direct benefit, high burden, low uncertainty): 81%; €300 [€140–300]. Scenario 2 (some direct benefit, low burden, moderate uncertainty): 48%; €150 [€100–200]. Scenario 3 (some direct benefit, high burden and high uncertainty): 60%; €500 [€200–1,000].

**Conclusion:**

Respondents in this exploratory French survey largely supported financial compensation for participation in drug clinical trials. Compensation is perceived as a proportionate ethical and social recognition of uncertainty, burden and lack of direct benefit. These findings provide an empirical basis for developing transparent tools to guide compensation decisions.

## Introduction

According to the European Clinical Trials Regulation (EU No 536/2014), “no incentives or financial inducements should be given to the subjects, except for compensation for expenses and loss of earnings directly related to participation in a clinical trial” [1]. This principle aims to protect participants from undue influence and to ensure that consent remains voluntary [2,3]. Financial compensation should reflect the inconvenience experienced by participants while avoiding both excessive and insufficient payment. Like the regulatory framework, ethical guidance from the Council of Europe states that compensation should remain proportionate to the burden or inconvenience associated with participation and should not constitute undue influence on participants’ decisions [4].

In practice, compensation is determined by sponsors and reviewed by ethics committees, yet no standardized or transparent framework exists to justify its necessity or amount. European regulations provide limited operational guidance, and although the Clinical Trials Coordination and Advisory Group has proposed a model compensation form, its categories remain broad and offer limited practical value, with vague categories such as “loss of earnings” or “other” [5].

In 2023, the INDEM working group (INDEM standing for “indemnisation”, the French term for “compensation”) was established to address patient compensation in interventional drug research. This French multidisciplinary group includes investigator physicians, ethicists, sponsors, sociologists, and health economists [6,7]. Initial observations revealed marked disparities between compensation offered to healthy volunteers—who are routinely compensated, sometimes reaching several thousand euros depending on study burden and duration—and patients, who often receive little or no financial recognition [6,7]. This discrepancy is frequently justified by assumptions that patients benefit from access to innovative treatments or that altruism alone should motivate participation [8–10].

To address these gaps, the INDEM working group aimed to determine criteria for compensation. Initially, a working group composed of medical investigators, ethics committee members, and a sociologist held a brainstorming session on the determinants of compensation in late 2023. The working group recommended gathering community perspectives before developing preliminary framework. The objective of the present study was exploratory, the aim was not to formally quantify preferences or estimate trade-offs between compensation attributes, but rather to obtain preliminary information on the acceptability of compensation, identify potentially relevant determinants, and explore how members of the public react to different clinical trial situations. The objective was to align future guidance with both ethical standards and public expectations. This study reports the findings of the INDEM3 survey, which explored public perceptions of financial compensation for patients involved in drug clinical trials and identified factors influencing these views.

## Materials and methods

### Study design and participants

As no prior quantitative data were available and the objective of the study was exploratory rather than hypothesis-testing, no formal sample size calculation was performed. As an exploratory study, the sample size was determined for semi‑structured interviews, thematic saturation is often reached between about 20 and 40 interviews; therefore 60 is justified to cover the population profile [11]. The sample size was therefore guided by feasibility considerations and the aim of capturing a broad range of perspectives rather than achieving statistical representativeness. In this study, community perspectives refer to structured quantitative responses from members of the general population rather than qualitative deliberative data. Although participants were asked to adopt a patient perspective in scenario-based questions, responses were analyzed as reflecting respondents’ own judgments regarding the acceptability and justification of financial compensation.

### Questionnaire and data collection

The questionnaire was developed through a multi-step process combining literature review and working group discussions.

#### Working group.

Initially, a working group composed of investigator physicians, ethics committee members, and a sociologist identified the main determinants potentially justifying financial compensation in drug clinical trials. Several recurring dimensions emerged from the literature and discussions, including perceived risk associated with investigational drugs, absence of direct benefit, invasive procedures, and time burden related to participation. Concerning risk, the working group considered that phase I to II clinical trials generally involve investigational treatments associated with higher perceived risk related to higher uncertainty, phase III with moderate perceived risk whereas phase IV trials involve drugs already used in routine clinical practice with lower risk related to lower uncertainty. This distinction was consistent with the European regulatory framework for low-intervention clinical trials.

These selected dimensions were intended to reflect ethically relevant aspects commonly considered in compensation decisions. Among all determinants discussed, the four most consistently retained for questionnaire development were: (1) uncertainty associated with a poorly known drug, (2) absence of direct benefit for the participant, (3) invasive procedures, and (4) time burden. Other factors discussed but not retained included advanced age, non-inferiority methodology, fear of adverse events, destabilization of a stable condition, and poor prognosis.

We also acknowledge that these dimensions reflect a simplified representation of real-world decision-making and do not capture the full complexity of clinical trial contexts.

Based on these dimensions, several hypothetical scenarios were developed using language accessible to the general public. The scenarios were inspired by ongoing clinical trials conducted at Caen Normandy University Hospital. To limit respondent burden, three scenarios were ultimately retained.

#### First version of the self-administered questionnaire.

The questionnaire was pilot-tested with five beta testers from the general population, none of whom were healthcare professionals, to assess clarity, comprehensibility, and face validity. The purpose of this pilot testing was to evaluate the wording, readability, and usability of the questionnaire rather than to revise or expand the predefined selection criteria, which had already been established by the multidisciplinary working group. Minor wording revisions were implemented based on their feedback before final dissemination.

#### Final version of the self-administered questionnaire.

The questionnaire was composed of three sections designed to gather sociodemographic information and explore participants’ perceptions and preferences regarding financial compensation for research participation. To address objectives, the questionnaire was decomposed in 3 steps: (step 1) prior to any explanation of the regulatory context, (step 2) after the regulatory framework was described, and (step 3) following the presentation of three illustrative scenarios.

**Step 1: (1):** Sociodemographic characteristics: data collected included age, sex, presence of a chronic illness, living arrangement (alone or not), socio-professional category, and self-reported household income.

Experience with clinical research: participants were asked about any previous involvement in clinical studies, their motivations for participating or reasons for declining, as well as the perceived benefits and drawbacks of such participation. Advantages and disadvantages about compensation in drug clinical trials were interrogated.

**Step 2:** Criteria for a compensation: multiple choice among 8 criteria defined after INDEM working group brainstorming and the possibility to add a new one.

**Step 3:** Illustrative scenarios: three hypothetical research situations (Table 1) were presented, varying in terms of level of constraint, uncertainty, and potential direct benefit to the participant. Respondents were asked whether, if placed in the same situation, they would have agreed to participate in the research, whether the participant should receive compensation, if so what amount and how did they estimate this amount (free text).

**Table 1:** Description of the 3 scenarios.

	Risk	Direct Benefit	Constraints	
			Additional procedures	Time
Scenario 1 The patient is an elderly man with chronic kidney failure who undergoes hemodialysis three times per week. Because his kidneys no longer function adequately, medications are not eliminated from his body in the usual way. His nephrologist invites him to take part in a research study designed to better understand how an anticoagulant drug is eliminated in patients receiving hemodialysis. This medication is already well established and routinely prescribed to patients without kidney failure. The aim of the study is to determine the appropriate dosage for hemodialysis patients who may need this anticoagulant in the future. The patient himself does not currently require this treatment. Participation in the study would involve a three-day hospital stay, including overnight stays. On the first day, the patient would take a single tablet of the anticoagulant. Blood samples would be collected over the three days through an intravenous catheter, and urine would also be collected during this period. His usual treatment and overall medical care would remain unchanged throughout the study.	Low uncertainty -Well known drug	No	Blood and urine collection	3 days of hospitalisation
Scenario 2 The patient is a middle-aged woman who has recently been diagnosed with breast cancer. Her oncologist invites her to participate in a research study evaluating a new chemotherapy regimen. This treatment is already well established and widely used for ovarian cancer, but its use in breast cancer is still being studied. Participation in the study would require two additional blood samples to be taken at the beginning of treatment.	Moderate uncertainty – established drug used in a new indication	Yes	Blood samples	None
Scenario 3 The patient is an elderly man who has been living with Parkinson’s disease for 30 years, and whose current treatment is no longer effective. His neurologist invites him to participate in a research study evaluating a new treatment currently in phase 2 of drug development. Participation would involve receiving a monthly infusion of the study drug at the hospital. In addition, the patient would be asked to undergo two lumbar punctures: the first after six months of treatment and the second after one year. His usual medical care would otherwise remain unchanged.	High uncertainty – investigational treatment	Yes	2 lumbar punctures 12 infusions	Monthly visits during one year

At the end of each step: General opinion on compensation: participants were asked whether they agreed or disagreed with the principle of financial compensation for research participation was assessed at the end of each steps. Evolution of their opinion across the 3 steps was evaluated.

#### Recruitment.

The self-administered questionnaire required approximately seven minutes to complete and was available in both paper and digital formats. Paper questionnaires were distributed in a university hospital diabetology department, a clinical research center, and a community pharmacy. The online version was disseminated via social networks to broaden reach.

### Ethics

The survey protocol and questionnaire (Supplementary Material 1) were approved by a local ethics committee (Comité Local d’Éthique de la Recherche en Santé, CHU de Caen Normandie et Normandie Université; ID 5069, February 2024). An information form was provided to all participants (Supplementary Material 2). In accordance with national regulations, informed consent was not required.

### Statistical analysis

Continuous parametric variables are presented as means with standard deviations and continuous non-parametric variables are presented as medians with first and third quartiles in square brackets. Categorical variables are expressed as numbers and percentages. Comparative analyses for chronic illness effect and for prior participation in research were done used chi-square or Fisher’s exact tests for small sample as well as percentages comparisons in respondents’ answers. Statistical significance was set at p ≤ 0.05. Analyses were performed using JASP version 0.19.3. Reporting results followed the Consensus-Based Checklist for Reporting of Survey Studies (CROSS) [12].

## Results

### Participant characteristics

A total of 65 responses were collected between June 27, 2024, and January 31, 2025: 22 (33.8%) in healthcare settings, and 43 (66.2%) via social networks. The characteristics of the population are presented in Table 2. No significant difference was observed in terms of socio-professional category between our sample and the 2022 French general population (p = 0.42) [13,14].

**Table 2:** Characteristics of the overall respondents.

	Overall respondents (n = 65)
**Age (years)**	49.1 ± 13.8
**Gender**	
Male	25 (38.4%)
Female	40 (61.5%)
**Chronic disease**	
Yes	23 (35.4%)
No	42 (64.6%)
**Socio-professional category**	
Executives and higher intellectual professions	12 (18.5%)
Intermediate level professions	9 (13.8%)
Employees or manual workers	27 (41.5%)
Inactive	17 (26.1%)
**Origin of recruitment**	
Healthcare settings (hospital or pharmacy)	22 (33.8%)
Social network	43 (66.2%)
**Estimated income**	
High or very high (≥3900€/ month/ person)	14 (21.5%)
Medium or low (<3900€/ month/ person)	49 (75.4%)
Not answered	2 (3.1%)
**Previous solicitation for a participation to a clinical trial**	
Yes	26 (40%)
No	36 (55.4%)
Not answered	3 (4.6%)
**Previous participation to a clinical trial among those sollicitated**	**(n = 26)**
Yes	22(84.6%)
No	4 (15.4%)

Continuous variables are described by the mean and standard deviation. Categorical variables are described by the number of respondents and the corresponding percentage (%).

### General views on compensation

Overall, a large majority of respondents supported financial compensation for participation in drug clinical trials, reaching 75% among 53 respondents with a final opinion. This level of support remained stable across steps (1) before any contextual information, (2) after the explanation of relevant guidelines, and (3) after reviewing three fictional scenarios (Table 3).

**Table 3:** Transition of opinions on financial compensation across the 3 steps of the questionnaire.

In favor of compensation for participants	Yes	No	No answer	n=
At step 1: general naïve opinion	54 (83.1%)	3 (4.6%)	8 (12.3%)	65
After step 2: after explanation of relevant guideline and suggestion of determinants	57 (87.7%)	3 (4.6%)	5 (7.7%)	65
After step 3: opinion after thinking about scenarios where they projected themselves in the place of the participants in a clinical trial	49 (75.4%)	4 (6.1%)	12 (18.5%)	65

Participants with chronic illnesses were significantly less likely to support compensation at steps two and three compared to those without chronic conditions (87% (20/23) vs. 100%(37/37), p = 0.02 and 77.8% (14/18) vs. 100% (35/35), p = 0.004, respectively). Prior participation in research was not associated with different views toward compensation (94.4% (17/18) versus 100% (4/4) p = 0.63 at step 1, 82.6% (19/20) versus 100% (4/4); p = 0.65 at step 2, and 94.4% (17/18) versus 100% (4/4) p = 0.63 at step 3).

### Perceived advantages and disadvantages of research participation without context (Step 1)

When asked about the perceived advantages and disadvantages of participating in clinical trials, the most frequently cited benefit was advancing care for other patients, while the most commonly reported disadvantage was the potential risk of taking new treatments involved (Table 4).

**Table 4:** Perceived advantages and disadvantages of participating in a clinical trial (n = 65).

		Selected (Yes), n(%)	Not selected, n(%)
Advantages	Advancing the care of other patients	58 (89.2%)	7 (10.8%)
	Advantage: Benefit from the latest scientific advances	46 (70.8%)	19 (29.2%)
	Other:	2 (3.1%) -relieve doctors -less painful care	63 (96.9%)
Disadvantages	Disadvantage: Taking risks with new treatments	45 (69.2%)	20 (30.8%)
	Disadvantage: Spending more time in the hospital	31 (47.7%)	34 (52.3%)
	Disadvantage: Having more medical procedures	24 (36.9%)	41 (63.1%)
	Other	2 (3.1%) -loss of earnings being forced to take a day off (1) -unknow new procedures (1)	63 (76.9%)

### Justification criteria for compensation after the explanation of relevant guidelines (step 2)

Participants were presented with seven predefined criteria that could justify financial compensation and were given the opportunity to suggest additional ones (Table 5). The most frequently endorsed criteria were: taking a new medication (n = 42; 64.6%), undergoing invasive or traumatic procedures (n = 41; 63.1%), and experiencing income loss (n = 41; 63.1%). No additional criteria beyond the seven initially proposed were suggested by participants.

**Table 5:** Criteria justifying compensation.

Criteria for compensation	Yes	No	No answer	n=
Taking a new medication whose effects are still poorly or little known	42 (64.6%)	22 (33.8%)	1 (1.5%)	65
Invasive-traumatic examinations	41 (63.1%)	23 (35.4%)	1 (1.5%)	65
Losing income due to time spent in hospital rather than at work	41 (63.1%)	23 (35.4%)	1 (1.5%)	65
Additional days of hospitalization	34 (52.3%)	30 (46.2%)	1 (1.5%)	65
Consultations in addition to your usual follow-up	22 (33.9%)	42 (64.6%)	1 (1.5%)	65
Not having a direct benefit from participating in this clinical trial	18 (27.7%)	46 (70.8%)	1 (1.5%)	65
Receiving medication intravenously rather than orally	16 (24.6%)	48 (73.9%)	1 (1.5%)	65
Taking a placebo	12 (18.5%)	52 (80%)	1 (1.5%)	65
Criteria for compensation Other	0 (0%)	0 (0%)	65 (100%)	65

### Responses to drug clinical trial scenarios after reviewing three fictional scenarios (step 3)

Participants were asked to place themselves in the role of a patient in three fictional trial scenarios and indicate whether they would participate depending on whether compensation was offered. Multiple responses were allowed (Table 6).

**Table 6:** Willingness to participate in each scenario. Scenario 1: absence of direct benefit, high burden, and low uncertainty. Scenario 2: low burden, potential direct benefit, and moderate uncertainty with exposure to an established drug used in a new indication. Scenario 3: high burden, potential direct benefit, and high uncertainty with exposure to a new drug.

Agreement to participate to the clinical trial regarding the compensation	Scenario	Yes	No	No answer	n=
Would have participated if compensated	1	47 (72.3%)	14 (21.5%)	4 (6.2%)	65
	2	34 (52.3%)	25 (38.5%)	6 (9.2%)	65
	3	37 (56.9%)	22 (33.9%)	6 (9.2%)	65
Would have participated without compensation	1	18 (27.7%)	43 (66.1%)	4 (6.2)%	65
	2	36 (55.4%)	23 (35.4%)	6 (9.2%)	65
	3	20 (30.8%)	39 (60.0%)	6 (9.2%)	65
Would have declined if compensated	1	3 (4.6%)	58 (89.2%)	4 (6.2%)	65
	2	3 (4.6%)	56 (86.2%)	6 (9.2%)	65
	3	4 (6.2%)	55 (84.6%)	6 (9.2%)	65
Would have declined without compensation	1	3 (4.6%)	58 (89.2%)	4 (6.2%)	65
	2	3 (4.6%)	56 (86.2%)	6 (9.2%)	65
	3	10 (15.4%)	49 (75.4%)	6 (9.2%)	65

Consistency toward compensation was observed between scenarios (Table 6).

In scenario 1, defined by the absence of direct benefit, high burden, and low uncertainty with a well-known drug, majority (n = 47; 72.3%) of participants would have participated if compensated but also if not compensated (n = 43; 66.1%). They mainly answered that they would not have declined regardless of compensation (n = 58; 89.2%). In this scenario, (n = 53; 81.5%) of participants supported financial compensation, and proposed a median amount of €300 [140–300] for patients’ participation.

In scenario 2 characterized by low burden, potential direct benefit, and moderate uncertainty with exposure to an established drug used in a new indication, respondents would have participated if compensated but in a smaller proportion (n = 34; 52.3%) than in scenario 1 (p < 0.001) and most would have accepted to participate even if there were no compensation (n = 36; 55.4%). In this scenario, (n = 31) 47.7% supported compensation with a median proposed amount of €150 [100–200].

In Scenario 3, involving high burden, potential direct benefit, and high uncertainty with exposure to a new drug, majority of respondents (n = 37; 56.9%) would have participate if compensated, and majority of respondents (n = 39; 60%) would not agree to participate without compensation.

In this scenario, (n = 39; 60%) of participants supported compensation, with a median proposed amount of €500 [200–1000].

Overall, participants justified the proposed compensation amounts through free-text explanations. Creating a word cloud highlights these 8 key words: risk/effects (n = 13), hospital/hospitalization (n = 9), medication/treatment (n = 9), time/constraints (n = 7), travel (n = 4), puncture/invasive/additional (n = 8), earnings/work (n = 5), progress of medicine (n = 4).

## Discussion

### Public support for compensation

This survey suggests a high level of support for financial compensation of drug clinical trial participants. The results of this survey are consistent with previous European and Anglo-Saxon studies, which report that the majority of participants consider it fair to receive financial compensation for participating in clinical research [15–17]. These convergences suggest that the expectations observed are not specific to the French context, but reflect a broader and shared understanding of the ethical issues associated with research participation. The present study explored public perceptions of financial compensation broadly understood and did not attempt to formally distinguish reimbursement, compensation for burden, and potential incentive effects as defined in regulatory frameworks.

### Criteria for compensation

Participants identified several relevant criteria for justifying compensation, including the uncertainty of exposure to new drugs, the burden of procedures (especially invasive ones), time spent (e.g., hospitalization or additional visits), and the absence of direct benefit. These criteria reflect an intuitive understanding of both burden and risk and align with prior studies examining public and patient perspectives on research participation [8–10]. Devlin et al. reported a semi-structured interview of 58 potential research participants that mentioned that participant should be compensated for time, effort and risk [8]. A 2024 review of the current literature on factors influencing patients’ decisions to participate in clinical trials identified ten key factors. Among these, four factors specifically relate to the burden of participation: side effects, the perception of being a “guinea pig”, the required effort and time commitment, and the use of a placebo [9]. The criteria collected here may thus serve to inform evidence-based thresholds for future tools.

### Views of patients with chronic conditions

Patients with chronic conditions seem less attached to financial compensation. The OECD’s PaRIS survey provides insights that could explain these differences with non-patients, pointing to a better overall experience of the healthcare system among individuals with chronic illnesses. In France, nearly all people with chronic conditions report receiving good quality care (91%), which is higher than the OECD average in the PaRIS survey (around 85%) [18].

### Amounts

Suggested amounts from public ranged from €150 for studies involving minimal burden to €500 for studies involving the highest level of burden.. Overall, these amounts appear modest and unlikely to constitute undue inducement, particularly in light of the constraints and risks associated with research participation. For comparison, the UK Health Research Authority recommends a minimum compensation of £200 per study day(23). In France, a decree issued in February 2023 increased the annual ceiling for participant compensation to €6,000, indexed to inflation, thereby acknowledging the legitimacy of higher compensation levels while maintaining regulatory safeguards. Taken together, these findings suggest that participants’ expectations remain well below existing regulatory thresholds, supporting the ethical acceptability of financial compensation in clinical research.

### Ethical considerations

#### Compensation and altruism.

Contrary to the belief that financial incentives might undermine altruism, our findings suggest that compensation can coexist with altruistic motives. The most frequently cited reason for participation was “helping to improve care for other patients,” ahead of personal benefits such as access to innovative treatments. While French bioethics traditionally favors strict altruism—as in the case of organ and gamete donation—evidence from other studies indicates that financial recognition does not necessarily diminish altruistic intent. For example, most oocyte donors in Europe receive compensation even though altruism remains their primary motivation [19]. These findings support the idea that offering compensation acknowledges participants’ contributions without negating their altruism.

#### Risk and absence of direct benefit.

The survey reveals a nuanced public view regarding risk. While guidelines typically allow compensation for inconvenience and time—but not for risk—risk (or uncertainty) was frequently cited as a factor associated with justification of compensation by respondents. For instance, the CIOMS Guidelines exclude risk-based payments [20], whereas Australian and UK guidelines are more permissive, highlighting the need for flexibility [21,22]. Respondents appeared to react primarily to the perceived novelty and uncertainty associated with investigational drugs. Moreover, participants consistently emphasized the absence of direct benefit as a standalone criterion. Although this concept is largely absent from regulatory frameworks, it intuitively resonates with public expectations and could be considered an implicit dimension of risk.

Compensation is then perceived not merely as an incentive, but as a form of ethical and social recognition of the time commitment, burden, and risks associated with participation [15–17,23,24].

#### Transparency.

Compensation may help clarify the distinction between research-related and therapeutic procedures, thereby reducing the risk of therapeutic misconception when transparently explained during informed consent [25,26]. The UK royal College of Physicians’Guidelines on the practice of ethics committees in medical research with human participants [27] argues that payment may help patients distinguish procedures that are done purely for research purposes from those done for their benefit, thus minimising vulnerability due to « therapeutic misconception » [25]. Compensation should therefore be a payment conceptualized as a transparent reward for the participant’s contribution to social beneficence. Cryder et al. found in US that high participation payments increased willingness to participate, but, consistent with the idea that people infer riskiness from payment amount, high payments also increased perceived risk and time spent viewing risk information and conclude that discrepancy between research guidelines and participants’ assumptions about those guidelines has implications for informed consent in human subjects research [25]. The information form should use clear and comprehensive language to ensure a good understanding of the compensation process and enhance overall study comprehension [28].

#### Ethical obligation to compensate.

According to several authors, failing to offer compensation may constitute a form of ethical exploitation [15,29,30]. Research participants provide a social service by contributing to clinical research, often accepting measurable risks and burdens without any direct personal benefit. The absence of compensation may discourage participation and disproportionately favor individuals who are able to absorb the associated costs, thereby reinforcing social inequalities.

### Recruitment

From a public perspective, absence of compensation may discourage participation and contribute to social inequalities in access to research [15–17,31]. Previous studies highlighted the importance of compensation in recruitment [26]. According to the survey, in the case of the scenario without direct benefit, the inclusion could be increased with compensation, underscoring the significant impact of financial incentives on participation decisions. The recent review on patients’ motivators and barriers to clinical trial participation identified ten key factors, including financial compensation [32]. This supports the idea that understanding motivators can serve as a lever to accelerate patient enrolment, ultimately influencing the overall speed of recruitment—a critical aspect of any clinical trial. In the same way, a quality study in 2022, among 37 participants in randomized clinical trials, indicated that money had been a motivation for enrolling but did not use reasoning that suggested undue influence, unjust inducement, or therapeutic misconception. In this study, Largent et al. then encourage IRBs to relax restrictions on use of incentives in trials [33].

### Perspectives

In this perspective, the findings of the present study provide a solid empirical basis for the development of decision-support tools for sponsors and ethics committees, allowing to:

- standardize compensation according to uncertainty, burden, and absence of direct benefit
- ensure clear and transparent information for participants regarding the rationale and calculation of compensation;
- enhance equity and reduce socio-economic barriers to participation in clinical research.

The originality of this study lies in its structured scenario-based approach combined with quantitative evaluation of proposed compensation amounts. This design provides empirical insight into public reasoning and identifies concrete criteria that could inform the development of transparent decision-support tools for sponsors and ethics committees.

### Limitations

Several limitations should be acknowledged. First, the exploratory cross-sectional design does not allow causal inference or preference modelling. Recruitment via social network may have introduced a selection bias, favoring individuals already engaged with health or research topics. In addition, the limited sample size did not allow robust subgroup analyses according to recruitment modality (clinical settings versus social media recruitment). Second, hypothetical scenarios may not fully capture the emotional and contextual complexity of real-world decision-making [19,24]. Third, the study was conducted in France, with respondents predominantly from middle- and upper-socioeconomic backgrounds, which may limit the generalizability of the findings to other populations. Then, the wording and selection of questionnaire items may have introduced framing effects and influenced respondents’ judgments regarding compensation. Finally, the questionnaire specifically focused on drug clinical trials, and the findings may therefore not be generalizable to other forms of clinical research, which participants may perceive differently.

## Conclusion

Participants in this exploratory French survey largely supported proportionate financial compensation for drug clinical trial participants, recognizing the ethical and social value of their contribution and providing a basis for transparent guidance.

### Take-home message

Transparent and proportionate financial compensation is ethically supported by public opinion and should recognize the contributions of all drug clinical trial participants.

## Supporting information

S1 File Questionnaire. (DOCX)
