# Supplementary material for: Ethical and social recognition of participation in drug clinical trials: French public views on financial compensation for participants
Source: PLoS One. 2026 Aug 24;21(8):e0343321. doi: 10.1371/journal.pone.0343321 (PMC13502642; doi:10.1371/journal.pone.0343321)
Supplement: Supplementary file 1 [file pone.0343321.s001.docx]

Questionnaire

**INDEM3**

**COMMUNITY QUESTIONNAIRE**
**STUDY ON "PATIENT COMPENSATION IN CLINICAL TRIALS: A USER OPINION SURVEY"**

In France today, patients who agree to participate in drug-related clinical research may receive compensation. However, our previous studies have shown that this practice is inconsistent, and in most cases, patients do not receive any compensation for their participation.

The INDEM3 team, which is working on this issue, aims to clarify the situations in which compensation should be provided.

We believe that the first step in this process is to gather opinions from patients who might be affected by such research.

It is important to note that in all clinical research, patients are systematically reimbursed for direct costs such as travel expenses and meals.
However, they are not always financially compensated.

By answering this questionnaire, you will help us understand the perspective of voluntary patient participants.
First, we will ask for your general opinion, then we will present you with three realistic scenarios to assess your viewpoint.

**Do you agree to complete this questionnaire?**
☐ YES / ☐ NO
If no, why? __________

**Your Socio-Professional Characteristics and General Opinion**

**Age:** _____ years
**Gender:** ☐ Male ☐ Female

**Do you have a chronic illness?**
☐ No
☐ Yes. If yes, which one? __________

**Do you have someone in your household who helps you manage your illness?**
☐ Yes
☐ No

**Do you live alone?**
☐ Yes
☐ No

**What is your socio-professional category?**
☐ Farmers
☐ Craftsmen
☐ Traders
☐ Business owners
☐ Executives and intellectual professions
☐ Intermediate professions
☐ Employees
☐ Workers
☐ Retired
☐ Student
☐ Unemployed

**Among these options—low, medium, high, very high—which best describes your household income?**
☐ Low
☐ Medium
☐ High
☐ Very high

**Have you ever been invited to participate in a clinical trial?**
☐ Yes
☐ No

**If yes, did you accept?**
☐ Yes
☐ No

**If no, what prevented you from participating?**

**If yes:**

- What motivated you to participate?
- Was the research related to your illness? ☐ Yes ☐ No
- Did you receive compensation? ☐ Yes ☐ No
  - If yes, in what form? ☐ Cash ☐ Gift voucher ☐ Other: __________
  - If no, would you have liked to receive compensation for your participation? ☐ Yes ☐ No

**From your perspective, what are the benefits of participating in research?** (multiple answers possible)
☐ Better medical care
☐ Access to the latest scientific advancements
☐ Contributing to the improvement of care for other patients
☐ Other: __________

**From your perspective, what are the disadvantages of participating in research?**
☐ Spending more time in the hospital
☐ Undergoing more medical procedures
☐ Taking risks with new treatments
☐ Other: __________

**Would you support financial compensation for patients participating in research?**
☐ Yes
☐ No
☐ Not sure

**Your Opinion on Compensation Criteria and Amount**

Ethical guidelines state:

*"The ethics committee must ensure that any payment or compensation given to participants is appropriate to the burden or inconvenience caused by the research but not so significant that it unduly influences their decision to participate."*

Due to these vague definitions, participants are rarely compensated. By gathering your perspective, we aim to clarify the criteria for determining compensation and its amount.

**Which criteria should be considered when deciding on compensation?** (multiple answers possible)
☐ Additional hospitalization days
☐ Additional medical visits beyond routine care
☐ Invasive/traumatic procedures (e.g., colonoscopy)
☐ Receiving an intravenous drug instead of an oral drug
☐ Loss of income due to time spent at the hospital instead of work
☐ Taking a new drug with unknown or poorly understood effects
☐ Taking a placebo
☐ No direct benefit from participating (e.g., testing a drug for a condition one does not have)
☐ Other, specify: __________

**Overall, based on the criteria you selected, do you support compensating patients in clinical drug research?**
☐ Yes
☐ No

**Case Studies**

We ask you to put yourself in the place of the patients described in the following three scenarios.

**Case 1**

A 70-year-old man with chronic kidney failure undergoes dialysis three times a week. His nephrologist invites him to participate in a study assessing the elimination of an anticoagulant in dialysis patients. The drug is well-known and widely used in non-dialysis patients. The study will help determine appropriate dosing for dialysis patients who may need this anticoagulant in the future (though he does not currently need it).

To participate, he must stay in the hospital for three days, including overnight stays. He will take a single dose of the anticoagulant on the first day, and his blood will be drawn via an intravenous catheter over three days. His urine will also be collected during this period. His usual treatment and care remain unchanged.

Should he receive compensation? (multiple answers possible)
☐ No, participation is a privilege
☐ No, his altruism benefits the medical community
☐ Yes, for the inconvenience of a three-day hospital stay
☐ Yes, for the blood draws and urine collection
☐ Yes, for the risk of side effects from the drug
☐ Yes, as he receives no direct personal benefit

Would you participate in his place?
☐ Yes, if compensated
☐ Yes, without compensation
☐ No, if compensated
☐ No, without compensation

**If compensated, in what form?** ☐ Cash ☐ Gift voucher ☐ Other: __________

**What amount should he receive?** _______€

**How did you estimate this amount?**

**Case 2**

A 25-year-old woman has just been diagnosed with breast cancer. Her oncologist invites her to participate in a six-month study on a new chemotherapy, already known to be effective for ovarian cancer. The only difference from standard care is two additional blood tests at the beginning of treatment.

Should she receive compensation? (multiple answers possible)
☐ No, it’s an opportunity for her to try this medication
☐ No, her participation benefits medical research
☐ Yes, for the inconvenience of two extra blood tests
☐ Yes, for the risk of side effects from a new drug

Would you participate in her place?
☐ Yes, if compensated
☐ Yes, without compensation
☐ No, if compensated
☐ No, without compensation

**Case 3**

An 82-year-old man has had Parkinson’s disease for 30 years and no longer responds to treatment. His neurologist invites him to join a study on a new drug (still largely unknown). He would receive a monthly intravenous infusion at the hospital for one year and undergo two lumbar punctures (one at six months and one at one year).

Should he receive compensation? (multiple answers possible)
☐ No, participation is a privilege
☐ No, it benefits the medical community
☐ Yes, for the inconvenience of monthly hospital visits
☐ Yes, for the infusions
☐ Yes, for the lumbar punctures
☐ Yes, for the risk of side effects

Would you participate in his place?
☐ Yes, if compensated
☐ Yes, without compensation
☐ No, if compensated
☐ No, without compensation

**Final Question**

**Overall, do you support compensating patients who participate in clinical research?**
☐ Yes
☐ No
