# Supplementary material for: Ethical and social recognition of participation in drug clinical trials: French public views on financial compensation for participants
Source: PLoS One. 2026 Aug 24;21(8):e0343321. doi: 10.1371/journal.pone.0343321 (PMC13502642; doi:10.1371/journal.pone.0343321)
Supplement: Supplementary file 2 [file pone.0343321.s003.docx]

Point-by-point responses

1. Please ensure that your manuscript meets PLOS ONE's style requirements, including those for file naming. The PLOS ONE style templates can be found at

<https://journals.plos.org/plosone/s/file?id=wjVg/PLOSOne_formatting_sample_main_body.pdf> and

<https://journals.plos.org/plosone/s/file?id=ba62/PLOSOne_formatting_sample_title_authors_affiliations.pdf>

2. We note that two of the authors of the present submission (Guillaume GRANDAZZI, Rémy MORELLO) is listed as members of the ethics committee on the approval document provided. Could you please explain what your ethical committee's policies are when an Author is also a member of the committee, and how this was handled in this specific case? Thank you very much for your response to this request.

Authors: Although Guillaume GRANDAZZI and Rémy MORELLO are members of the ethics committee, they were not involved in the evaluation, discussion, or decision-making process regarding this specific study. In accordance with the ethics committee’s internal policies on conflicts of interest, any member who is directly involved in a submitted project is excluded from the review and approval procedure for that project.

The protocol was therefore independently reviewed and approved by the remaining members of the committee, without participation from the authors concerned.

3. In the online submission form you indicate that your data is not available for proprietary reasons and have provided a contact point for accessing this data. Please note that your current contact point is a co-author on this manuscript. According to our Data Policy, the contact point must not be an author on the manuscript and must be an institutional contact, ideally not an individual. Please revise your data statement to a non-author institutional point of contact, such as a data access or ethics committee, and send this to us via return email. Please also include contact information for the third party organization, and please include the full citation of where the data can be found.

If the reviewer comments include a recommendation to cite specific previously published works, please review and evaluate these publications to determine whether they are relevant and should be cited. There is no requirement to cite these works unless the editor has indicated otherwise.

Authors: We have revised the Data Availability Statement accordingly. The dataset will be made available upon reasonable request through the Data Management Unit of Caen Normandy University Hospital (CHU Caen Normandie), which is independent from the authorship of the manuscript.

Revised Data Availability Statement:

“The data underlying the results presented in this study are not publicly available due to legal and ethical restrictions related to participant confidentiality. Data are available upon reasonable request from the Data Management Unit, CHU Caen Normandie, Avenue de la Côte de Nacre, 14033 Caen Cedex 9, France, RC-Datamanager@chu-caen.fr.”

Additional Editor Comments:

Please use the (TWO) referee reports to improve your paper. At this stage, I don't know if the revised version will have any chance of being accepted (rejection is possible, even with the revised version); but it is clear that the draft article needs improvement in any case.

Authors: We carefully considered all comments provided by both reviewers.

**Reviewer #1**

Reviewer #1: The authors present original research regarding community views of payments for research participation in the context of interventional drug research. The goal is to inform recommendations for researchers and research ethics committees. This research could add to data in other countries.

While what is presented is presented clearly, there are details that are missing that raise questions about the methods and the strength of the interpretations. Specifically:
1. I would encourage the authors to summarize qualitatively in the text the kinds of questions that the participants were asked in terms of opinions about compensation and about the scenarios.

Authors: Thank you for this helpful suggestion. We agree that a clearer qualitative description of the questionnaire content improves the readability of the Methods section. We have therefore expanded the manuscript to provide a more detailed overview of the questionnaire structure.

2. I would also encourage the authors to provide more details regarding the development of the scenarios and the questionnaire. Was there any pre-testing of the scenarios or the questionnaire? How were the questionnaire options (e.g., for criteria for compensation) selected? Are they supported in the literature? Answers to these questions could address some concerns I have. For example, at line 246, there is a statement that "Respondents did not distinguish between different stages of drug development" but it is not clear that respondents had an understanding of these phases or could discern them in the scenarios.

Thank you for this important comment. We agree that additional details regarding the development of the questionnaire and scenarios were necessary to better support interpretation of the findings.

We therefore expanded both the Introduction and the Methods sections to clarify how the questionnaire and scenarios were developed, how the compensation criteria were selected, and how distinctions between different types of drug clinical trials were operationalized in the scenarios.

We also revised the Discussion to avoid overinterpreting respondents’ perceptions of drug development stages. Respondents were not asked to identify formal clinical trial phases, nor was knowledge of trial methodology assessed. Rather, the scenarios were intentionally constructed to represent different levels of perceived uncertainty, burden, and expected benefit. The interpretation of respondents’ answers has therefore been limited to these characteristics rather than to specific clinical trial phases.

3. I would suggest presenting the data for the two recruitment groups, in addition to the overall sample, as it seems likely (but I cannot tell) that the group recruited within clinical settings would look different from those recruited from social media.

Authors: Thank you for this relevant suggestion. We agree that recruitment modality may influence participant characteristics and perceptions. We have therefore added the recruitment source information to the table describing participant characteristics.

However, because of the limited sample size, particularly after stratification according to recruitment modality, we considered that subgroup analyses would not be sufficiently powered to provide robust or interpretable comparisons between participants recruited in clinical settings and those recruited through social media. We have therefore chosen not to perform formal subgroup analyses and acknowledge this as a limitation of the study.

4. The regulations quoted at lines 56-58 distinguishes between the impermissible "incentives" and "inducements" and the permissible "compensation for expenses or loss of earnings." This is different from the ethical guidelines that are provided in the questionnaire. What is the source of the ethical guidelines, and why was this language selected over the regulatory language for the questionnaire?

Authors: Thank you for this important request for clarification. The wording used in the questionnaire was derived from the “Guide for Research Ethics Committee Members” published by the Council of Europe in the context of the Oviedo Convention on biomedical research.

The relevant statement appears in section 6.C.22.1 (“Participants”):

“The REC should be satisfied that any payments and rewards to participants are appropriate to the burden or inconvenience caused by the research and are not of such magnitude as to lead participants to take risks that they would not otherwise take.”

Council of Europe. Guide for Research Ethics Committee Members. Strasbourg: Council of Europe; 2011.

This reference has now been added to the Introduction section of the manuscript and is conceptually consistent with the regulatory framework cited in the manuscript.

We selected this formulation because it reflects the ethical reasoning commonly used by research ethics committees when evaluating participant compensation, particularly the balance between fair compensation and the avoidance of undue influence. Although the terminology differs somewhat from the regulatory distinction between “compensation” and “inducement,” both approaches are conceptually aligned in aiming to prevent inappropriate financial influence on participation decisions.

5. I would also encourage the authors add to the limitations section that the questionnaire was limited to drug trials, as participants may view them differently from other research.

Authors: Thank you for this relevant suggestion. We agree that perceptions regarding financial compensation may differ depending on the type of research involved.

To clarify the scope of the study, we have modified the title to explicitly specify that the study concerns “drug clinical trials.” In addition, we have expanded the Limitations section to state that the findings cannot necessarily be generalized to other types of clinical research or non-drug studies, as participants may perceive these contexts differently.

**Reviewer #2**

Reviewer #2: This manuscript addresses a relevant ethical and methodological issue, namely the justification of financial compensation for participation in clinical drug research. However, despite the interest of the topic, the study in its current form presents substantial weaknesses in conceptual framing, methodological design, questionnaire construction, analysis, and reporting, which prevent a reliable interpretation of the findings.
Given these limitations, I deliberately do not go beyond a descriptive assessment of the results. The current introduction and methodological framework are insufficiently specified to support more substantive interpretation or policy relevant conclusions. Substantial revision would be required before the manuscript could be adequately evaluated.

Authors: The revised manuscript now includes a dedicated subsection describing the development of the questionnaire. The dimensions explored were identified through a combination of literature review and multidisciplinary discussions involving investigator physicians, ethics committee members, a sociologist, and other members of the INDEM working group. The selected dimensions were intended to represent relevant factors frequently discussed in relation to compensation decisions, including uncertainty associated with investigational treatments, procedural invasiveness, time burden, and absence of direct benefit.

We acknowledge that the wording of some items may have influenced respondents’ perceptions. Accordingly, we now explicitly recognize potential framing effects as a limitation of the study. We also clarify that the scenarios were designed as simplified hypothetical situations intended to explore reactions to selected attributes rather than to reproduce the full complexity of real-world clinical trial decision-making.

1. Conceptual framing and positioning in the literature
The introduction does not sufficiently situate the study within the existing empirical and ethical literature on compensation in clinical research.
• The term clinical trial is used in a broad and undifferentiated manner, without clarification regarding trial phase, level of risk, type of intervention, pathology, or target population, although these factors are central to compensation practices and ethical debates.

We thank the reviewer for this important observation and agree that the initial version of the manuscript insufficiently specified the scope and heterogeneity of clinical trials.

To address this point, we revised the title and the introduction to clarify that the present study specifically focuses on interventional drug clinical trials. We also added explicit regarding the importance of uncertainty, burden of participation, and expected direct benefit, as these dimensions are central to ethical debates surrounding compensation.

In addition, the three fictional scenarios were designed to reflect varying combinations of uncertainty, burden, and direct benefit in order to better capture how these dimensions may influence public views regarding compensation.

We believe these revisions better situate the study within the ethical and empirical literature on compensation in clinical research and clarify the specific context explored in this survey.

• References to existing observations or practices remain vague and are not supported by concrete empirical data (e.g. usual ranges of compensation).

Authors: To address this point, we revised the Introduction to include concrete observations from previous French studies on compensation practices in clinical research.

• Concepts with strong interpretative implications (e.g. community perspectives, patient empowerment) are invoked without clear definition or operationalization, making their relevance to the present design unclear.
Overall, the research question remains insufficiently precise and weakly anchored in the literature.

Authors: We agree that the initial version of the manuscript did not sufficiently clarify key conceptual distinctions related to clinical trial characteristics (e.g., phase, risk level, and intervention type) and compensation practices. The title and the introduction have therefore been revised to more precisely define the scope of “drug clinical trials”.

We have also clarified and operationalized key concepts such as “community perspectives” by explicitly linking them to survey-based public opinion data in the context of clinical research ethics. In addition, references to compensation practices and regulatory frameworks have been strengthened with more explicit contextualization of ethical guidelines and European regulatory texts.

2. Study design
2.1 Sampling strategy
The methodological rationale for the study design is insufficiently developed.
• The absence of sample size calculation is justified solely by the lack of prior data, which is not methodologically convincing. Sample size should be justified in relation to the study design and analytical objectives, not only data availability.

• The stated aim of “capturing diversity of perspectives” is not clearly linked to either the chosen sample size (n=60) or a defined sampling strategy.

Authors: We agree that alternative approaches such as qualitative interviews, focus groups, mixed-methods designs, or stated-preference techniques could have provided a more detailed understanding of participants’ reasoning and preference structures.

However, the objective of the present study was not to formally model preferences, estimate trade-offs between attributes, or generate population-representative estimates. Rather, this exploratory survey was designed as an initial step within the INDEM project to document public reactions to the issue of financial compensation, identify potentially relevant dimensions, and generate empirical data in an area where little information is currently available in the French context.

Within this exploratory framework, a structured questionnaire combined with simple hypothetical scenarios was considered an appropriate and pragmatic approach. This design allowed the collection of quantitative information on attitudes toward compensation, perceived justifications, and proposed compensation amounts across several clinical research contexts.

We have revised the manuscript to more clearly emphasize the exploratory nature of the study and to avoid interpretations that would exceed the methodological scope of the design.

• The decision to include one third of participants living with a chronic disease is not adequately justified, and the relevance of chronic disease as a criterion in this context is not discussed.
Authors: Thank you for this comment. We agree that the wording in the original manuscript may have suggested that a predefined proportion of participants living with chronic disease was planned. This was not the case.

The study did not use quota sampling, and no target proportion of participants with chronic disease was specified a priori. Individuals with and without chronic disease were eligible for participation, and recruitment was conducted in both healthcare settings and through social networks in order to obtain a diversity of perspectives and experiences.

We have revised the manuscript to remove any suggestion that one-third of participants with chronic disease was a predefined recruitment objective. The observed proportion of respondents living with chronic disease reflects the characteristics of the participants who chose to respond rather than a planned sampling criterion.

2.2 Questionnaire construction and framing effects
The overall study design does not appear well aligned with the stated objective of exploring preferences, justifications, and reasoning underlying compensation judgments. In addition, given the nature of the research question—aiming to explore preferences, justifications, and reasoning underlying compensation judgments—it is unclear why neither a qualitative approach nor a more formally structured quantitative method was adopted. In particular, the manuscript does not explain why an exploratory qualitative design (e.g. interviews or focus groups), a mixed methods approach, or a stated preference framework such as a discrete choice experiment or contingent valuation was not considered. This absence of justification further weakens the coherence between the research question, the chosen design, and the type of conclusions drawn.

We thank the reviewer for this important observation. We agree that qualitative methods, mixed-methods approaches, or stated-preference techniques such as discrete choice experiments (DCEs) may provide a deeper understanding of the reasoning processes underlying judgments about financial compensation.

The objective of the present study was, however, more modest and exploratory. At this stage of the INDEM project, our aim was not to formally quantify preferences or estimate trade-offs between compensation attributes, but rather to obtain preliminary information on the acceptability of compensation, identify potentially relevant determinants, and explore how members of the public react to different clinical trial situations.

The questionnaire, which constitutes the core methodological instrument of the study, is insufficiently justified and raises concerns about framing and induction effects. It is not specified how response options were developed (e.g. based on the literature, a pilot study, qualitative pre work, or expert consultation). Several questions implicitly assume the presence of risk, burden, or financial loss (e.g. “taking risks with new treatments”, “loss of income”), although these are dependent on the type and phase of clinical trials and are not systematic features of participation. Given this framing, it is unsurprising that for example “taking risks with new treatments” emerges as the most frequent justification for compensation.
The construction of the scenarios further compounds these issues. Respondents are described as being asked to put themselves “in the place of the patient”, yet the questionnaire alternates between questions referring to a fictional patient and questions referring to the respondent’s own position, creating conceptual confusion about the perspective being elicited. In addition, response options allow multiple selections combining affirmative and negative answers, and questions concerning willingness to participate oneself are conflated with judgments about compensating participants. Together, these inconsistencies significantly undermine the interpretability of the responses.

Authors: We agree that the framing of questionnaire items required clearer justification. The revised manuscript now explains that the selected dimensions were identified through both literature review and multidisciplinary expert discussions, and were intended to reflect relevant aspects commonly considered in compensation decisions, including burden, procedural invasiveness, uncertainty related to investigational treatments, and absence of direct benefit.

3. Analysis, presentation of results and discussion
Criteria for using means or medians are not specified, and no analytical model is proposed. The structure of the Results section is also problematic: the paragraph on lines 138–142, referring to “analyses of associated factors”, appears at the very beginning of the Results section and should come after a clear descriptive presentation of the data.
Moreover, the manuscript lacks a clear and structured results table summarizing the number of respondents for each item and each response option. Given the small sample size, transparency regarding denominators is essential. Similarly, figures do not systematically report sample sizes. Categories representing less than 5% of respondents are reported separately, which may overemphasize sparse data and hinder interpretation; regrouping rare categories would improve clarity.
An additional concern is that the questionnaire includes the question “How did you estimate this amount?”, which I do not found in the Results section. This question could have provided crucial insight into respondents’ reasoning and reference points.

Taken together, the limitations in study design, questionnaire construction, and analytical strategy are substantial. In their current form, these weaknesses are too significant to allow reliable interpretation of the findings or to support publication.

We agree that greater transparency in reporting was necessary. The Methods section has been revised to clarify the use of descriptive statistics, including the rationale for using means versus medians depending on distribution.

We have also:

•	reorganized the Results section to ensure a clearer descriptive flow before inferential analyses,

•	added or clarified denominators in all tables and converted some figures into tables for clarity, to systematically report sample sizes

•	and reviewed categorical groupings to avoid over-interpretation of very small categories.

Regarding the question “How did you estimate this amount?”, we acknowledge that this item was included in the questionnaire but not reported in the Results. We have now added this analysis in the Results section.

We recognize that the initial version of the manuscript required additional methodological detail and clarification in several areas. We have therefore substantially revised the manuscript to strengthen the conceptual framing, improve transparency regarding questionnaire development and sampling rationale, and enhance the coherence between the study objectives, design, analyses, and interpretation of findings.

We believe these revisions substantially improve the clarity, methodological transparency, and interpretability of the study.
