# Supplementary material for: Ethical and social recognition of participation in drug clinical trials: French public views on financial compensation for participants
Source: PLoS One. 2026 Aug 24;21(8):e0343321. doi: 10.1371/journal.pone.0343321 (PMC13502642; doi:10.1371/journal.pone.0343321)
Supplement: Supplementary file 3 [file pone.0343321.s004.docx]

Point-by-point responses

We sincerely thank the reviewer for his careful reading of our revised manuscript and for his constructive comments. We appreciate the opportunity to further improve our work. Below, we provide a point-by-point response to each comment. All modifications have been incorporated into the revised manuscript and are highlighted in the tracked version.

Reviewer #1: I appreciate the authors' responsiveness to the reviewers' comments. I have some remaining questions, as well as some new ones posed by the revisions. (Note: my line references refer to the tracked version)

Methods questions:
Lines 93-97. The explanation is appreciated, but I would move this explanation to the methods (around lines 103-111).

Thank you for this helpful suggestion. We agree that the rationale for the methodological approach is better placed in the Methods section. Accordingly, we have moved the explanation from the Introduction to the Methods section.

In this introductory section, I would like to see the explanation for choosing to take a quantitative approach over a qualitative approach (as requested by reviewer 2), as qualitative approaches are generally preferred for exploratory studies.

In the Introduction, we now explicitly justify our choice of a quantitative approach. “The objective of the present study was exploratory, the aim was not to formally quantify preferences or estimate trade-offs between compensation attributes, but rather to obtain preliminary information on the acceptability of compensation, identify potentially relevant determinants, and explore how members of the public react to different clinical trial situations.”

Lines 103-11. Again, the explanation is appreciated, but the explanation lacks support. Is there any support in the literature, for example, to suggest that the target sample size would achieve the heterogeneity that the authors indicate they sought?

Thank you for this valuable comment. We agree that the rationale for our target sample size required additional support. To address this point, we have revised the Methods section to include a literature-based justification.

Lines 120-170. The additional information helps me better understand the process, but I still have questions about the beta testing. From the description, it seems that there was a focus on the language of the questions rather than the content. Given the exploratory nature of this study, was there any attempt during this beta testing to check whether the testers, who were drawn from the general population, identified criteria or other potential questions that were different from the ones that the researchers and working group had identified?

Thank you for this important comment. The objective of the beta testing was not to identify additional selection criteria but to evaluate the clarity, comprehensibility, and usability of the questionnaire before dissemination. The predefined criteria had already been established by the multidisciplinary working group during the questionnaire development process and were therefore not intended to be modified during the pilot testing. To avoid any ambiguity, we have clarified this objective in the Methods section.

Substantive questions:
Lines 43, 50-51 of the abstract change the start date of data collection (from June 2024 to March) and the percentages for Scenarios 2 and 3. I did not see an explanation for these changes in the authors' response.

Thank you for noting this inconsistency. These values were corrected following verification of the final dataset after revision. We apologize for not explicitly mentioning these corrections in our previous response letter.

Line 218 has another change in reported percentage without an explanation.

Thank you for identifying this oversight. This percentage was also updated following verification of the final analyses. We apologize for failing to explicitly report this correction in our previous response letter.

The revised manuscript has been checked to ensure all reported values are now consistent.

Table 4, Based on the table, it appears that potential responses were "Yes" or "No answer". Is that accurate? If not, I would recommend editing to present actual response choices.

Thank you for this helpful comment. You are correct that participants could either select a proposed criterion or leave it unselected; there was no explicit "No" response option. We agree that the label "No answer" could be misleading, as it may suggest missing data rather than an intentionally unselected criterion. To improve clarity, we have revised the table headings to "Selected (Yes), n (%)" and "Not selected, n (%)", which more accurately reflect the response options available to participants.

Suggested edits:
Line 68, The beginning phase "In the same way. . . " is awkward. I would suggest a simpler phrase such as "Like the regulatory framework."

Thank you for this suggestion. We have replaced this expression with "Like the regulatory framework."

Table 2, 1) I would recommend that the authors add the income ranges that would show how they define income levels. 2) I find the insertion of the data of those who have actually participated in clinical trials into the responses on prior recruitment confusing. I would suggest moving it back to a separate cell, but note that n=26 rather than full sample.

Thank you for these helpful suggestions.

Income ranges have been added to improve clarity.

Previous participation in clinical trials has been presented separately (n = 26), as suggested.

Line 238, I would suggest adding a description of what Step 1 is (i.e., without context) for clarity. I would continue to provide that explanation when moving to Step 2 and 3.

Thank you. We agree that this improves readability. We have added a brief description of each step when first introduced and maintained this approach for Steps 2 and 3.

Table 6, As some readers will look to the tables without the accompanying text, I would encourage the authors to organize the table so that there is a brief description of each Scenario.

Thank you for this suggestion. Brief descriptions of each scenario have now been added to Table 6.

Lines 291-292, I could not discern what the first sentence meant. I would rephrase.

Thank you. The sentence has been rephrased to improve clarity.

Line 338, the word "maximal" needs context.

Thank you for this helpful comment. We agree that the term "maximal" was too vague. We have revised the sentence to explicitly refer to the study burden, replacing "the maximal" with ""studies involving the highest level of burden”, to improve clarity.

We thank the reviewer again for these thoughtful comments, which have helped us improve the clarity, transparency, and overall quality of the manuscript.
