# Supplementary material for: Ethical and social recognition of participation in drug clinical trials: French public views on financial compensation for participants
Source: PLoS One. 2026 Aug 24;21(8):e0343321. doi: 10.1371/journal.pone.0343321 (PMC13502642; doi:10.1371/journal.pone.0343321)
Supplement: Peer review history

# Peer review: Decision Letter 0

**DOI:** 10.1371/journal.pone.0343321.r001

**Authors:** Bruno Ventelou

12 May 2026

Dear Dr. Peyro-Saint-Paul,

Thank you for submitting your manuscript to PLOS ONE. After careful consideration, we feel that it has merit but does not fully meet PLOS ONE’s publication criteria as it currently stands. Therefore, we invite you to submit a revised version of the manuscript that addresses the points raised during the review process.

Please submit your revised manuscript by Jun 26 2026 11:59PM. If you will need more time than this to complete your revisions, please reply to this message or contact the journal office at plosone@plos.org. When you're ready to submit your revision, log on to https://www.editorialmanager.com/pone/ and select the 'Submissions Needing Revision' folder to locate your manuscript file.

- A letter that responds to each point raised by the academic editor and reviewer(s). You should upload this letter as a separate file labeled 'Response to Reviewers'.
- A marked-up copy of your manuscript that highlights changes made to the original version. You should upload this as a separate file labeled 'Revised Manuscript with Track Changes'.
- An unmarked version of your revised paper without tracked changes. You should upload this as a separate file labeled 'Manuscript'.

If you would like to make changes to your financial disclosure, please include your updated statement in your cover letter. Guidelines for resubmitting your figure files are available below the reviewer comments at the end of this letter.

If applicable, we recommend that you deposit your laboratory protocols in protocols.io to enhance the reproducibility of your results. Protocols.io assigns your protocol its own identifier (DOI) so that it can be cited independently in the future. For instructions see: https://journals.plos.org/plosone/s/submission-guidelines#loc-laboratory-protocols. Additionally, PLOS ONE offers an option for publishing peer-reviewed Lab Protocol articles, which describe protocols hosted on protocols.io. Read more information on sharing protocols at https://plos.org/protocols?utm_medium=editorial-email&utm_source=authorletters&utm_campaign=protocols.

As the corresponding author, your ORCID iD is verified in the submission system and will appear in the published article. PLOS supports the use of ORCID, and we encourage all coauthors to register for an ORCID iD and use it as well. Please encourage your coauthors to verify their ORCID iD within the submission system before final acceptance, as unverified ORCID iDs will not appear in the published article. *Only*  the individual author can complete the verification step; PLOS staff *cannot*  verify ORCID iDs on behalf of authors.

We look forward to receiving your revised manuscript.

Kind regards,

Bruno Ventelou

Academic Editor

PLOS One

Journal requirements:

When submitting your revision, we need you to address these additional requirements.

1. Please ensure that your manuscript meets PLOS ONE's style requirements, including those for file naming. The PLOS ONE style templates can be found at

https://journals.plos.org/plosone/s/file?id=wjVg/PLOSOne_formatting_sample_main_body.pdf and

https://journals.plos.org/plosone/s/file?id=ba62/PLOSOne_formatting_sample_title_authors_affiliations.pdf

2. We note that two of the authors of the present submission (Guillaume GRANDAZZI, Rémy MORELLO) is listed as members of the ethics committee on the approval document provided. Could you please explain what your ethical committee's policies are when an Author is also a member of the committee, and how this was handled in this specific case? Thank you very much for your response to this request.

3. In the online submission form you indicate that your data is not available for proprietary reasons and have provided a contact point for accessing this data. Please note that your current contact point is a co-author on this manuscript. According to our Data Policy, the contact point must not be an author on the manuscript and must be an institutional contact, ideally not an individual. Please revise your data statement to a non-author institutional point of contact, such as a data access or ethics committee, and send this to us via return email. Please also include contact information for the third party organization, and please include the full citation of where the data can be found.

If the reviewer comments include a recommendation to cite specific previously published works, please review and evaluate these publications to determine whether they are relevant and should be cited. There is no requirement to cite these works unless the editor has indicated otherwise.

Additional Editor Comments:

Please use the (TWO) referee reports to improve your paper. At this stage, I don't know if the revised version will have any chance of being accepted (rejection is possible, even with the revised version); but it is clear that the draft article needs improvement in any case.

[Note: HTML markup is below. Please do not edit.]

Reviewers' comments:

Reviewer's Responses to Questions

**Comments to the Author**

1. Is the manuscript technically sound, and do the data support the conclusions?

Reviewer #1: Partly

Reviewer #2: No

**********

2. Has the statistical analysis been performed appropriately and rigorously?

Reviewer #1: I Don't Know

Reviewer #2: I Don't Know

**********

3. Have the authors made all data underlying the findings in their manuscript fully available?

The PLOS Data policy

Reviewer #1: No

Reviewer #2: No

**********

4. Is the manuscript presented in an intelligible fashion and written in standard English?

Reviewer #1: Yes

Reviewer #2: Yes

**********

Reviewer #1: The authors present original research regarding community views of payments for research participation in the context of interventional drug research. The goal is to inform recommendations for researchers and research ethics committees. This research could add to data in other countries.

While what is presented is presented clearly, there are details that are missing that raise questions about the methods and the strength of the interpretations. Specifically:

1. I would encourage the authors to summarize qualitatively in the text the kinds of questions that the participants were asked in terms of opinions about compensation and about the scenarios.

2. I would also encourage the authors to provide more details regarding the development of the scenarios and the questionnaire. Was there any pre-testing of the scenarios or the questionnaire? How were the questionnaire options (e.g., for criteria for compensation) selected? Are they supported in the literature? Answers to these questions could address some concerns I have. For example, at line 246, there is a statement that "Respondents did not distinguish between different stages of drug development" but it is not clear that respondents had an understanding of these phases or could discern them in the scenarios.

3. I would suggest presenting the data for the two recruitment groups, in addition to the overall sample, as it seems likely (but I cannot tell) that the group recruited within clinical settings would look different from those recruited from social media.

4. The regulations quoted at lines 56-58 distinguishes between the impermissible "incentives" and "inducements" and the permissible "compensation for expenses or loss of earnings." This is different from the ethical guidelines that are provided in the questionnaire. What is the source of the ethical guidelines, and why was this language selected over the regulatory language for the questionnaire?

5. I would also encourage the authors add to the limitations section that the questionnaire was limited to drug trials, as participants may view them differently from other research.

Reviewer #2: This manuscript addresses a relevant ethical and methodological issue, namely the justification of financial compensation for participation in clinical drug research. However, despite the interest of the topic, the study in its current form presents substantial weaknesses in conceptual framing, methodological design, questionnaire construction, analysis, and reporting, which prevent a reliable interpretation of the findings.

Given these limitations, I deliberately do not go beyond a descriptive assessment of the results. The current introduction and methodological framework are insufficiently specified to support more substantive interpretation or policy relevant conclusions. Substantial revision would be required before the manuscript could be adequately evaluated.

1. Conceptual framing and positioning in the literature

The introduction does not sufficiently situate the study within the existing empirical and ethical literature on compensation in clinical research.

• The term clinical trial is used in a broad and undifferentiated manner, without clarification regarding trial phase, level of risk, type of intervention, pathology, or target population, although these factors are central to compensation practices and ethical debates.

• References to existing observations or practices remain vague and are not supported by concrete empirical data (e.g. usual ranges of compensation).

• Concepts with strong interpretative implications (e.g. community perspectives, patient empowerment) are invoked without clear definition or operationalization, making their relevance to the present design unclear.

Overall, the research question remains insufficiently precise and weakly anchored in the literature.

2. Study design

2.1 Sampling strategy

The methodological rationale for the study design is insufficiently developed.

• The absence of sample size calculation is justified solely by the lack of prior data, which is not methodologically convincing. Sample size should be justified in relation to the study design and analytical objectives, not only data availability.

• The stated aim of “capturing diversity of perspectives” is not clearly linked to either the chosen sample size (n=60) or a defined sampling strategy.

• The decision to include one third of participants living with a chronic disease is not adequately justified, and the relevance of chronic disease as a criterion in this context is not discussed.

2.2 Questionnaire construction and framing effects

The overall study design does not appear well aligned with the stated objective of exploring preferences, justifications, and reasoning underlying compensation judgments. In addition, given the nature of the research question—aiming to explore preferences, justifications, and reasoning underlying compensation judgments—it is unclear why neither a qualitative approach nor a more formally structured quantitative method was adopted. In particular, the manuscript does not explain why an exploratory qualitative design (e.g. interviews or focus groups), a mixed methods approach, or a stated preference framework such as a discrete choice experiment or contingent valuation was not considered. This absence of justification further weakens the coherence between the research question, the chosen design, and the type of conclusions drawn.

The questionnaire, which constitutes the core methodological instrument of the study, is insufficiently justified and raises concerns about framing and induction effects. It is not specified how response options were developed (e.g. based on the literature, a pilot study, qualitative pre work, or expert consultation). Several questions implicitly assume the presence of risk, burden, or financial loss (e.g. “taking risks with new treatments”, “loss of income”), although these are dependent on the type and phase of clinical trials and are not systematic features of participation. Given this framing, it is unsurprising that for example “taking risks with new treatments” emerges as the most frequent justification for compensation.

The construction of the scenarios further compounds these issues. Respondents are described as being asked to put themselves “in the place of the patient”, yet the questionnaire alternates between questions referring to a fictional patient and questions referring to the respondent’s own position, creating conceptual confusion about the perspective being elicited. In addition, response options allow multiple selections combining affirmative and negative answers, and questions concerning willingness to participate oneself are conflated with judgments about compensating participants. Together, these inconsistencies significantly undermine the interpretability of the responses.

3. Analysis, presentation of results and discussion

Criteria for using means or medians are not specified, and no analytical model is proposed. The structure of the Results section is also problematic: the paragraph on lines 138–142, referring to “analyses of associated factors”, appears at the very beginning of the Results section and should come after a clear descriptive presentation of the data.

Moreover, the manuscript lacks a clear and structured results table summarizing the number of respondents for each item and each response option. Given the small sample size, transparency regarding denominators is essential. Similarly, figures do not systematically report sample sizes. Categories representing less than 5% of respondents are reported separately, which may overemphasize sparse data and hinder interpretation; regrouping rare categories would improve clarity.

An additional concern is that the questionnaire includes the question “How did you estimate this amount?”, which I do not found in the Results section. This question could have provided crucial insight into respondents’ reasoning and reference points.

Taken together, the limitations in study design, questionnaire construction, and analytical strategy are substantial. In their current form, these weaknesses are too significant to allow reliable interpretation of the findings or to support publication.

**********

what does this mean?). If published, this will include your full peer review and any attached files.

If you choose “no”, your identity will remain anonymous but your review may still be made public.

**Do you want your identity to be public for this peer review?**  For information about this choice, including consent withdrawal, please see our Privacy Policy

Reviewer #1: No

Reviewer #2: No

**********

[NOTE: If reviewer comments were submitted as an attachment file, they will be attached to this email and accessible via the submission site. Please log into your account, locate the manuscript record, and check for the action link "View Attachments". If this link does not appear, there are no attachment files.]

To ensure your figures meet our technical requirements, please review our figure guidelines: https://journals.plos.org/plosone/s/figures

You may also use PLOS’s free figure tool, NAAS, to help you prepare publication quality figures: https://journals.plos.org/plosone/s/figures#loc-tools-for-figure-preparation.

NAAS will assess whether your figures meet our technical requirements by comparing each figure against our figure specifications.

# Author comment: Author response to Decision Letter 1

**DOI:** 10.1371/journal.pone.0343321.r002

10 Jun 2026

Point-by-point responses

1. Please ensure that your manuscript meets PLOS ONE's style requirements, including those for file naming. The PLOS ONE style templates can be found at

https://journals.plos.org/plosone/s/file?id=wjVg/PLOSOne_formatting_sample_main_body.pdf and

https://journals.plos.org/plosone/s/file?id=ba62/PLOSOne_formatting_sample_title_authors_affiliations.pdf

Done.

2. We note that two of the authors of the present submission (Guillaume GRANDAZZI, Rémy MORELLO) is listed as members of the ethics committee on the approval document provided. Could you please explain what your ethical committee's policies are when an Author is also a member of the committee, and how this was handled in this specific case? Thank you very much for your response to this request.

Authors: Although Guillaume GRANDAZZI and Rémy MORELLO are members of the ethics committee, they were not involved in the evaluation, discussion, or decision-making process regarding this specific study. In accordance with the ethics committee’s internal policies on conflicts of interest, any member who is directly involved in a submitted project is excluded from the review and approval procedure for that project.

The protocol was therefore independently reviewed and approved by the remaining members of the committee, without participation from the authors concerned.

3. In the online submission form you indicate that your data is not available for proprietary reasons and have provided a contact point for accessing this data. Please note that your current contact point is a co-author on this manuscript. According to our Data Policy, the contact point must not be an author on the manuscript and must be an institutional contact, ideally not an individual. Please revise your data statement to a non-author institutional point of contact, such as a data access or ethics committee, and send this to us via return email. Please also include contact information for the third party organization, and please include the full citation of where the data can be found.

If the reviewer comments include a recommendation to cite specific previously published works, please review and evaluate these publications to determine whether they are relevant and should be cited. There is no requirement to cite these works unless the editor has indicated otherwise.

Authors: We have revised the Data Availability Statement accordingly. The dataset will be made available upon reasonable request through the Data Management Unit of Caen Normandy University Hospital (CHU Caen Normandie), which is independent from the authorship of the manuscript.

Revised Data Availability Statement:

“The data underlying the results presented in this study are not publicly available due to legal and ethical restrictions related to participant confidentiality. Data are available upon reasonable request from the Data Management Unit, CHU Caen Normandie, Avenue de la Côte de Nacre, 14033 Caen Cedex 9, France, RC-Datamanager@chu-caen.fr.”

Additional Editor Comments:

Please use the (TWO) referee reports to improve your paper. At this stage, I don't know if the revised version will have any chance of being accepted (rejection is possible, even with the revised version); but it is clear that the draft article needs improvement in any case.

Authors: We carefully considered all comments provided by both reviewers.

Reviewer #1

Reviewer #1: The authors present original research regarding community views of payments for research participation in the context of interventional drug research. The goal is to inform recommendations for researchers and research ethics committees. This research could add to data in other countries.

While what is presented is presented clearly, there are details that are missing that raise questions about the methods and the strength of the interpretations. Specifically:

1. I would encourage the authors to summarize qualitatively in the text the kinds of questions that the participants were asked in terms of opinions about compensation and about the scenarios.

Authors: Thank you for this helpful suggestion. We agree that a clearer qualitative description of the questionnaire content improves the readability of the Methods section. We have therefore expanded the manuscript to provide a more detailed overview of the questionnaire structure.

2. I would also encourage the authors to provide more details regarding the development of the scenarios and the questionnaire. Was there any pre-testing of the scenarios or the questionnaire? How were the questionnaire options (e.g., for criteria for compensation) selected? Are they supported in the literature? Answers to these questions could address some concerns I have. For example, at line 246, there is a statement that "Respondents did not distinguish between different stages of drug development" but it is not clear that respondents had an understanding of these phases or could discern them in the scenarios.

Thank you for this important comment. We agree that additional details regarding the development of the questionnaire and scenarios were necessary to better support interpretation of the findings.

We therefore expanded both the Introduction and the Methods sections to clarify how the questionnaire and scenarios were developed, how the compensation criteria were selected, and how distinctions between different types of drug clinical trials were operationalized in the scenarios.

We also revised the Discussion to avoid overinterpreting respondents’ perceptions of drug development stages. Respondents were not asked to identify formal clinical trial phases, nor was knowledge of trial methodology assessed. Rather, the scenarios were intentionally constructed to represent different levels of perceived uncertainty, burden, and expected benefit. The interpretation of respondents’ answers has therefore been limited to these characteristics rather than to specific clinical trial phases.

3. I would suggest presenting the data for the two recruitment groups, in addition to the overall sample, as it seems likely (but I cannot tell) that the group recruited within clinical settings would look different from those recruited from social media.

Authors: Thank you for this relevant suggestion. We agree that recruitment modality may influence participant characteristics and perceptions. We have therefore added the recruitment source information to the table describing participant characteristics.

However, because of the limited sample size, particularly after stratification according to recruitment modality, we considered that subgroup analyses would not be sufficiently powered to provide robust or interpretable comparisons between participants recruited in clinical settings and those recruited through social media. We have therefore chosen not to perform formal subgroup analyses and acknowledge this as a limitation of the study.

4. The regulations quoted at lines 56-58 distinguishes between the impermissible "incentives" and "inducements" and the permissible "compensation for expenses or loss of earnings." This is different from the ethical guidelines that are provided in the questionnaire. What is the source of the ethical guidelines, and why was this language selected over the regulatory language for the questionnaire?

Authors: Thank you for this important request for clarification. The wording used in the questionnaire was derived from the “Guide for Research Ethics Committee Members” published by the Council of Europe in the context of the Oviedo Convention on biomedical research.

The relevant statement appears in section 6.C.22.1 (“Participants”):

“The REC should be satisfied that any payments and rewards to participants are appropriate to the burden or inconvenience caused by the research and are not of such magnitude as to lead participants to take risks that they would not otherwise take.”

Council of Europe. Guide for Research Ethics Committee Members. Strasbourg: Council of Europe; 2011.

This reference has now been added to the Introduction section of the manuscript and is conceptually consistent with the regulatory framework cited in the manuscript.

We selected this formulation because it reflects the ethical reasoning commonly used by research ethics committees when evaluating participant compensation, particularly the balance between fair compensation and the avoidance of undue influence. Although the terminology differs somewhat from the regulatory distinction between “compensation” and “inducement,” both approaches are conceptually aligned in aiming to prevent inappropriate financial influence on participation decisions.

5. I would also encourage the authors add to the limitations section that the questionnaire was limited to drug trials, as participants may view them differently from other research.

Authors: Thank you for this relevant suggestion. We agree that perceptions regarding financial compensation may differ depending on the type of research involved.

To clarify the scope of the study, we have modified the title to explicitly specify that the study concerns “drug clinical trials.” In addition, we have expanded the Limitations section to state that the findings cannot necessarily be generalized to other types of clinical research or non-drug studies, as participants may perceive these contexts differently.

Reviewer #2

Reviewer #2: This manuscript addresses a relevant ethical and methodological issue, namely the justification of financial compensation for participation in clinical drug research. However, despite the interest of the topic, the study in its current form presents substantial weaknesses in conceptual framing, methodological design, questionnaire construction, analysis, and reporting, which prevent a reliable interpretation of the findings.

Given these limitations, I deliberately do not go beyond a descriptive assessment of the results. The current introduction and methodological framework are insufficiently specified to support more substantive interpretation or policy relevant conclusions. Substantial revision would be required before the manuscript could be adequately evaluated.

Authors: The revised manuscript now includes a dedicated subsection describing the development of the questionnaire. The dimensions explored were identified through a combination of literature review and multidisciplinary discussions involving investigator physicians, ethics committee members, a sociologist, and other members of the INDEM working group. The selected dimensions were intended to represent relevant factors frequently discussed in relation to compensation decisions, including uncertainty associated with investigational treatments, procedural invasiveness, time burden, and absence of direct benefit.

We acknowledge that the wording of some items may have influenced respondents’ perceptions. Accordingly, we now explicitly recognize potential framing effects as a limitation of the study. We also clarify that the scenarios were designed as simplified hypothetical situations intended to explore reactions to selected attributes rather than to reproduce the full complexity of real-world clinical trial decision-making.

1. Conceptual framing and positioning in the literature

The introduction does not sufficiently situate the study within the existing empirical and ethical literature on compensation in clinical research.

• The term clinical trial is used in a broad and undifferentiated manner, without clarification regarding trial phase, level of risk, type of intervention, pathology, or target population, although these factors are central to compensation practices and ethical debates.

We thank the reviewer for this important observation and agree that the initial version of the manuscript insufficiently specified the scope and heterogeneity of clinical trials.

To address this point, we revised the title and the introduction to clarify that the present study specifically focuses on interventional drug clinical trials. We also added explicit regarding the importance of uncertainty, burden of participation, and expected direct benefit, as these dimensions are central to ethical debates surrounding compensation.

In addition, the three fictional scenarios were designed to reflect varying combinations of uncertainty, burden, and direct benefit in order to better capture how these dimensions may influence public views regarding compensation.

We believe these revisions better situate the study within the ethical and empirical literature on compensation in clinical research and clarify the specific context explored in this survey.

• References to existing observations or practices remain vague and are not supported by concrete empirical data (e.g. usual ranges of compensation).

Authors: To address this point, we revised the Introduction to include concrete observations from previous French studies on compensation practices in clinical research.

• Concepts with strong interpretative implications (e.g. community perspectives, patient empowerment) are invoked without clear definition or operationalization, making their relevance to the present design unclear.

Overall, the research question remains insufficiently precise and weakly anchored in the literature.

Authors: We agree that the initial version of the manuscript did not sufficiently clarify key conceptual distinctions related to clinical trial characteristics (e.g., phase, risk level, and intervention type) and compensation practices. The title and the introduction have therefore been revised to more precisely define the scope of “drug clinical trials”.

We have also clarified and operationalized key concepts such as “community perspectives” by explicitly linking them to survey-based public opinion data in the context of clinical research ethics. In addition, references to compensation practices and regulatory frameworks have been strengthened with more explicit contextualization of ethical guidelines and European regulatory texts.

2. Study design

2.1 Sampling strategy

The methodological rationale for the study design is insufficiently developed.

• The absence of sample size calculation is justified solely by the lack of prior data, which is not methodologically convincing. Sample size should be justified in relation to the study design and analytical objectives, not only data availability.

• The stated aim of “capturing diversity of perspectives” is not clearly linked to either the chosen sample size (n=60) or a defined sampling strategy.

Authors: We agree that alternative approaches such as qualitative interviews, focus groups, mixed-methods designs, or stated-preference techniques could have provided a more detailed understanding of participants’ reasoning and preference structures.

However, the objective of the present study was not to formally model preferences, estimate trade-offs between attributes, or generate population-representative estimates. Rather, this exploratory survey was designed as an initial step within the INDEM project to document public reactions to the issue of financial compensation, identify potentially relevant dimensions, and generate empirical data in an area where little information is currently available in the French context.

Within this exploratory framework, a structured questionnaire combined with simple hypothetical scenarios was considered an appropriate and pragmatic approach. This design allowed the collection of quantitative information on attitudes toward compensation, perceived justifications, and proposed compensation amounts across several clinical research contexts.

We have revised the manuscript to more clearly emphasize the exploratory nature of the study and to avoid interpretations that would exceed the methodological scope of the design.

• The decision to include one third of participants living with a chronic disease is not adequately justified, and the relevance of chronic disease as a criterion in this context is not discussed.

Authors: Thank you for this comment. We agree that the wording in the original manuscript may have suggested that a predefined proportion of participants living with chronic disease was planned. This was not the case.

The study did not use quota sampling, and no target propo

Attachment Submitted filename: Response_to_Reviewers_20260608.docx

# Peer review: Decision Letter 1

**DOI:** 10.1371/journal.pone.0343321.r003

**Authors:** Bruno Ventelou

15 Jul 2026

Dear Dr. Peyro-Saint-Paul,

Thank you for submitting your manuscript to PLOS ONE. After careful consideration, we feel that it has merit but does not fully meet PLOS ONE’s publication criteria as it currently stands. Therefore, we invite you to submit a revised version of the manuscript that addresses the points raised during the review process.

Please submit your revised manuscript by Aug 29 2026 11:59PM. If you will need more time than this to complete your revisions, please reply to this message or contact the journal office at plosone@plos.org. When you're ready to submit your revision, log on to https://www.editorialmanager.com/pone/ and select the 'Submissions Needing Revision' folder to locate your manuscript file.

- A letter that responds to each point raised by the academic editor and reviewer(s). You should upload this letter as a separate file labeled 'Response to Reviewers'.
- A marked-up copy of your manuscript that highlights changes made to the original version. You should upload this as a separate file labeled 'Revised Manuscript with Track Changes'.
- An unmarked version of your revised paper without tracked changes. You should upload this as a separate file labeled 'Manuscript'.

If you would like to make changes to your financial disclosure, please include your updated statement in your cover letter. Guidelines for resubmitting your figure files are available below the reviewer comments at the end of this letter.

If applicable, we recommend that you deposit your laboratory protocols in protocols.io to enhance the reproducibility of your results. Protocols.io assigns your protocol its own identifier (DOI) so that it can be cited independently in the future. For instructions see: https://journals.plos.org/plosone/s/submission-guidelines#loc-laboratory-protocols. Additionally, PLOS ONE offers an option for publishing peer-reviewed Lab Protocol articles, which describe protocols hosted on protocols.io. Read more information on sharing protocols at https://plos.org/protocols?utm_medium=editorial-email&utm_source=authorletters&utm_campaign=protocols.

As the corresponding author, your ORCID iD is verified in the submission system and will appear in the published article. PLOS supports the use of ORCID, and we encourage all coauthors to register for an ORCID iD and use it as well. Please encourage your coauthors to verify their ORCID iD within the submission system before final acceptance, as unverified ORCID iDs will not appear in the published article. *Only*  the individual author can complete the verification step; PLOS staff *cannot*  verify ORCID iDs on behalf of authors.

We look forward to receiving your revised manuscript.

Kind regards,

Bruno Ventelou

Academic Editor

PLOS One

Journal Requirements:

1. If the reviewer comments include a recommendation to cite specific previously published works, please review and evaluate these publications to determine whether they are relevant and should be cited. There is no requirement to cite these works unless the editor has indicated otherwise.

2. Please review your reference list to ensure that it is complete and correct. If you have cited papers that have been retracted, please include the rationale for doing so in the manuscript text, or remove these references and replace them with relevant current references. Any changes to the reference list should be mentioned in the rebuttal letter that accompanies your revised manuscript. If you need to cite a retracted article, indicate the article’s retracted status in the References list and also include a citation and full reference for the retraction notice.

Additional Editor Comments:

Please take in consideration the second round of comments by referee 1 (see below)

[Note: HTML markup is below. Please do not edit.]

Reviewer's Responses to Questions

**Comments to the Author**

Reviewer #1: (No Response)

**********

2. Is the manuscript technically sound, and do the data support the conclusions?

Reviewer #1: Partly

**********

3. Has the statistical analysis been performed appropriately and rigorously?

Reviewer #1: I Don't Know

**********

4. Have the authors made all data underlying the findings in their manuscript fully available?

The PLOS Data policy

Reviewer #1: No

**********

5. Is the manuscript presented in an intelligible fashion and written in standard English?

Reviewer #1: Yes

**********

Reviewer #1: I appreciate the authors' responsiveness to the reviewers' comments. I have some remaining questions, as well as some new ones posed by the revisions. (Note: my line references refer to the tracked version)

Methods questions:

Lines 93-97. The explanation is appreciated, but I would move this explanation to the methods (around lines 103-111). In this introductory section, I would like to see the explanation for choosing to take a quantitative approach over a qualitative approach (as requested by reviewer 2), as qualitative approaches are generally preferred for exploratory studies.

Lines 103-11. Again, the explanation is appreciated, but the explanation lacks support. Is there any support in the literature, for example, to suggest that the target sample size would achieve the heterogeneity that the authors indicate they sought?

Lines 120-170. The additional information helps me better understand the process, but I still have questions about the beta testing. From the description, it seems that there was a focus on the language of the questions rather than the content. Given the exploratory nature of this study, was there any attempt during this beta testing to check whether the testers, who were drawn from the general population, identified criteria or other potential questions that were different from the ones that the researchers and working group had identified?

Substantive questions:

Lines 43, 50-51 of the abstract change the start date of data collection (from June 2024 to March) and the percentages for Scenarios 2 and 3. I did not see an explanation for these changes in the authors' response.

Line 218 has another change in reported percentage without an explanation.

Table 4, Based on the table, it appears that potential responses were "Yes" or "No answer". Is that accurate? If not, I would recommend editing to present actual response choices.

Suggested edits:

Line 68, The beginning phase "In the same way. . . " is awkward. I would suggest a simpler phrase such as "Like the regulatory framework."

Table 2, 1) I would recommend that the authors add the income ranges that would show how they define income levels. 2) I find the insertion of the data of those who have actually participated in clinical trials into the responses on prior recruitment confusing. I would suggest moving it back to a separate cell, but note that n=26 rather than full sample.

Line 238, I would suggest adding a description of what Step 1 is (i.e., without context) for clarity. I would continue to provide that explanation when moving to Step 2 and 3.

Table 6, As some readers will look to the tables without the accompanying text, I would encourage the authors to organize the table so that there is a brief description of each Scenario.

Lines 291-292, I could not discern what the first sentence meant. I would rephrase.

Line 338, the word "maximal" needs context.

**********

what does this mean?). If published, this will include your full peer review and any attached files.

If you choose “no”, your identity will remain anonymous but your review may still be made public.

**Do you want your identity to be public for this peer review?**  For information about this choice, including consent withdrawal, please see our Privacy Policy

Reviewer #1: No

**********

[NOTE: If reviewer comments were submitted as an attachment file, they will be attached to this email and accessible via the submission site. Please log into your account, locate the manuscript record, and check for the action link "View Attachments". If this link does not appear, there are no attachment files.]

To ensure your figures meet our technical requirements, please review our figure guidelines: https://journals.plos.org/plosone/s/figures

You may also use PLOS’s free figure tool, NAAS, to help you prepare publication quality figures: https://journals.plos.org/plosone/s/figures#loc-tools-for-figure-preparation.

NAAS will assess whether your figures meet our technical requirements by comparing each figure against our figure specifications.

# Author comment: Author response to Decision Letter 2

**DOI:** 10.1371/journal.pone.0343321.r004

20 Jul 2026

Point-by-point responses

We sincerely thank the reviewer for his careful reading of our revised manuscript and for his constructive comments. We appreciate the opportunity to further improve our work. Below, we provide a point-by-point response to each comment. All modifications have been incorporated into the revised manuscript and are highlighted in the tracked version.

Reviewer #1: I appreciate the authors' responsiveness to the reviewers' comments. I have some remaining questions, as well as some new ones posed by the revisions. (Note: my line references refer to the tracked version)

Methods questions:

Lines 93-97. The explanation is appreciated, but I would move this explanation to the methods (around lines 103-111).

Thank you for this helpful suggestion. We agree that the rationale for the methodological approach is better placed in the Methods section. Accordingly, we have moved the explanation from the Introduction to the Methods section.

In this introductory section, I would like to see the explanation for choosing to take a quantitative approach over a qualitative approach (as requested by reviewer 2), as qualitative approaches are generally preferred for exploratory studies.

In the Introduction, we now explicitly justify our choice of a quantitative approach. “The objective of the present study was exploratory, the aim was not to formally quantify preferences or estimate trade-offs between compensation attributes, but rather to obtain preliminary information on the acceptability of compensation, identify potentially relevant determinants, and explore how members of the public react to different clinical trial situations.”

Lines 103-11. Again, the explanation is appreciated, but the explanation lacks support. Is there any support in the literature, for example, to suggest that the target sample size would achieve the heterogeneity that the authors indicate they sought?

Thank you for this valuable comment. We agree that the rationale for our target sample size required additional support. To address this point, we have revised the Methods section to include a literature-based justification.

Lines 120-170. The additional information helps me better understand the process, but I still have questions about the beta testing. From the description, it seems that there was a focus on the language of the questions rather than the content. Given the exploratory nature of this study, was there any attempt during this beta testing to check whether the testers, who were drawn from the general population, identified criteria or other potential questions that were different from the ones that the researchers and working group had identified?

Thank you for this important comment. The objective of the beta testing was not to identify additional selection criteria but to evaluate the clarity, comprehensibility, and usability of the questionnaire before dissemination. The predefined criteria had already been established by the multidisciplinary working group during the questionnaire development process and were therefore not intended to be modified during the pilot testing. To avoid any ambiguity, we have clarified this objective in the Methods section.

Substantive questions:

Lines 43, 50-51 of the abstract change the start date of data collection (from June 2024 to March) and the percentages for Scenarios 2 and 3. I did not see an explanation for these changes in the authors' response.

Thank you for noting this inconsistency. These values were corrected following verification of the final dataset after revision. We apologize for not explicitly mentioning these corrections in our previous response letter.

Line 218 has another change in reported percentage without an explanation.

Thank you for identifying this oversight. This percentage was also updated following verification of the final analyses. We apologize for failing to explicitly report this correction in our previous response letter.

The revised manuscript has been checked to ensure all reported values are now consistent.

Table 4, Based on the table, it appears that potential responses were "Yes" or "No answer". Is that accurate? If not, I would recommend editing to present actual response choices.

Thank you for this helpful comment. You are correct that participants could either select a proposed criterion or leave it unselected; there was no explicit "No" response option. We agree that the label "No answer" could be misleading, as it may suggest missing data rather than an intentionally unselected criterion. To improve clarity, we have revised the table headings to "Selected (Yes), n (%)" and "Not selected, n (%)", which more accurately reflect the response options available to participants.

Suggested edits:

Line 68, The beginning phase "In the same way. . . " is awkward. I would suggest a simpler phrase such as "Like the regulatory framework."

Thank you for this suggestion. We have replaced this expression with "Like the regulatory framework."

Table 2, 1) I would recommend that the authors add the income ranges that would show how they define income levels. 2) I find the insertion of the data of those who have actually participated in clinical trials into the responses on prior recruitment confusing. I would suggest moving it back to a separate cell, but note that n=26 rather than full sample.

Thank you for these helpful suggestions.

Income ranges have been added to improve clarity.

Previous participation in clinical trials has been presented separately (n = 26), as suggested.

Line 238, I would suggest adding a description of what Step 1 is (i.e., without context) for clarity. I would continue to provide that explanation when moving to Step 2 and 3.

Thank you. We agree that this improves readability. We have added a brief description of each step when first introduced and maintained this approach for Steps 2 and 3.

Table 6, As some readers will look to the tables without the accompanying text, I would encourage the authors to organize the table so that there is a brief description of each Scenario.

Thank you for this suggestion. Brief descriptions of each scenario have now been added to Table 6.

Lines 291-292, I could not discern what the first sentence meant. I would rephrase.

Thank you. The sentence has been rephrased to improve clarity.

Line 338, the word "maximal" needs context.

Thank you for this helpful comment. We agree that the term "maximal" was too vague. We have revised the sentence to explicitly refer to the study burden, replacing "the maximal" with ""studies involving the highest level of burden”, to improve clarity.

We thank the reviewer again for these thoughtful comments, which have helped us improve the clarity, transparency, and overall quality of the manuscript.

Attachment Submitted filename: Response_to_Reviewers_20260720.docx

# Editor report: Decision Letter 2

**DOI:** 10.1371/journal.pone.0343321.r005

**Authors:** Bruno Ventelou

22 Jul 2026

Ethical and social recognition of participation in drug clinical trials: French public views on financial compensation for participants

PONE-D-26-05749R2

Dear Dr. Peyro-Saint-Paul,

We’re pleased to inform you that your manuscript has been judged scientifically suitable for publication and will be formally accepted for publication once it meets all outstanding technical requirements.

Within one week, you’ll receive an e-mail detailing the required amendments. When these have been addressed, you’ll receive a formal acceptance letter and your manuscript will be scheduled for publication.

An invoice will be generated when your article is formally accepted. Please note, if your institution has a publishing partnership with PLOS and your article meets the relevant criteria, all or part of your publication costs will be covered. Please make sure your user information is up-to-date by logging into Editorial Manager at Editorial Manager® and clicking the ‘Update My Information' link at the top of the page. For questions related to billing, please contact billing support.

If your institution or institutions have a press office, please notify them about your upcoming paper to help maximize its impact. If they’ll be preparing press materials, please inform our press team as soon as possible -- no later than 48 hours after receiving the formal acceptance. Your manuscript will remain under strict press embargo until 2 pm Eastern Time on the date of publication. For more information, please contact onepress@plos.org.

Kind regards,

Bruno Ventelou

Academic Editor

PLOS One

Additional Editor Comments (optional):

Reviewers' comments:

# Editor report: Acceptance letter

**DOI:** 10.1371/journal.pone.0343321.r006

**Authors:** Bruno Ventelou

PONE-D-26-05749R2

PLOS One

Dear Dr. Peyro-Saint-Paul,

I'm pleased to inform you that your manuscript has been deemed suitable for publication in PLOS One. Congratulations! Your manuscript is now being handed over to our production team.

At this stage, our production department will prepare your paper for publication. This includes ensuring the following:

* All references, tables, and figures are properly cited

* All relevant supporting information is included in the manuscript submission,

* There are no issues that prevent the paper from being properly typeset

You will receive further instructions from the production team, including instructions on how to review your proof when it is ready. Please keep in mind that we are working through a large volume of accepted articles, so please give us a few days to review your paper and let you know the next and final steps.

Lastly, if your institution or institutions have a press office, please let them know about your upcoming paper now to help maximize its impact. If they'll be preparing press materials, please inform our press team within the next 48 hours. Your manuscript will remain under strict press embargo until 2 pm Eastern Time on the date of publication. For more information, please contact onepress@plos.org.

You will receive an invoice from PLOS for your publication fee after your manuscript has reached the completed accept phase. If you receive an email requesting payment before acceptance or for any other service, this may be a phishing scheme. Learn how to identify phishing emails and protect your accounts at https://explore.plos.org/phishing.

If we can help with anything else, please email us at customercare@plos.org.

Thank you for submitting your work to PLOS ONE and supporting open access.

Kind regards,

PLOS ONE Editorial Office Staff

on behalf of

Dr. Bruno Ventelou

Academic Editor

PLOS One
